# FAR-AM: A hybrid attention framework for fire cause classification

**DOI:** 10.1371/journal.pone.0333131

**Published:** 2025-10-09

**Authors:** Heng Peng, Kun Zhu

**Affiliations:** 1 School of Management, China University of Mining and Technology (Beijing), Beijing, China; 2 Smart City Division, Shanxi Academy of Aerospace Technology Application Co., Ltd., Xi’an, Shanxi, China; Philadelphia University, JORDAN

## Abstract

Automated cause classification of fire accident reports (FIREAR) is crucial for enhancing public safety and developing data-driven prevention strategies. However, existing deep learning models often struggle with the unique challenges these documents present—namely their extreme length, high semantic noise, and fragmented causal information. To overcome these limitations, we propose the Fire Accident Reports Attention Mechanism (FAR-AM), a novel hybrid deep learning framework. FAR-AM first uses a large language model (LLM) to preprocess lengthy raw reports into concise, high-signal summaries. Its core architecture then employs an inter-layer self-attention mechanism to dynamically fuse hierarchical features across all encoder layers of BERT. The fused features are subsequently processed by a TextCNN for final classification. We evaluate FAR-AM on AGNews(title), AGNews(content), THUCNews, and our real-world FIREAR corpus. FAR-AM outperforms strong transformer baselines, including RoBERTa. On the FIREAR dataset, it achieves 73.58% accuracy and 70.65% F1. A comprehensive ablation study further validates the contribution of each component in the multi-stage framework. These results indicate that, for complex domain-specific tasks, specialized hybrid architectures can be more effective and robust than monolithic, general-purpose models.

## 1 Introduction

Fire accidents have become frequent and widespread disasters that threaten global public safety and pose serious risks to human lives and property [1]. These events not only cause direct harm to people but also have a profound social and economic impact. Therefore, improving fire emergency management capabilities is of paramount importance. A critical yet challenging aspect of this endeavor is the in-depth analysis of past accident investigation reports. By intelligently identifying the root causes of incidents from these textual records, fire departments can refine preventive measures and optimize the allocation of emergency resources. While many reporting systems are now structured, they invariably contain extensive unstructured narrative text detailing the incident’s process and causal analysis. This narrative text, rich with vital information but laden with unique complexities, presents a significant bottleneck for automated processing and is the central focus of this study.

The primary difficulty in automating the analysis of FIREAR stems from their inherent structural and semantic challenges, which are distinct from general-purpose text classification tasks. These reports are typically lengthy and structurally complex, often exceeding 12,000 words, and combine layered official formats with domain-specific terminology. Crucially, the causal factors are often fragmented and scattered across various administrative, operational, and technical sections. This fragmentation means that critical clues may be buried within vast amounts of redundant narrative and procedural descriptions, creating significant semantic complexity and noise that complicates automated analysis, a well-recognized challenge in robust text classification [2]. Consequently, any effective classification model must not only parse long-range dependencies but also discern and connect subtle, dispersed pieces of evidence to determine the primary cause.

Existing machine learning and deep learning approaches [3], while powerful, exhibit significant limitations when faced with these specific challenges, a topic of ongoing research in the broader field of text classification with transformers [5]. Traditional models like Support Vector Machines (SVM) or basic Recurrent Neural Networks (RNNs) often fail to capture the deep semantic context required [6]. Even advanced models like Bidirectional Encoder Representations from Transformers (BERT) have been sub-optimally applied [7]. The common practice of relying solely on the final layer’s output risks losing the granular, low-level feature representations from intermediate layers. This is a critical missed opportunity, as the success of multi-layer approaches in other complex domains highlights their potential [8,9]. While LLMs show promise, using them for end-to-end classification of such long documents is computationally expensive and can be inefficient at pinpointing fragmented causal links amidst noise, a concern echoed in research on parameter-efficient model tuning [10]. The culmination of these issues often leads to models with limited generalization capabilities for such specialized tasks [11]. These multifaceted limitations form a significant research gap and lead to our central research question: how can we design a model that effectively integrates deep, hierarchical features from pre-trained models to overcome the specific challenges of fragmented causality and semantic noise in FIREAR.

To address this gap, we propose the FAR-AM, an automatic text classification model specifically engineered for the complexities of FIREAR. Our hybrid architecture systematically tackles the aforementioned challenges. First, to manage the extreme length and narrative noise, we leverage an LLM not for classification, but as a sophisticated preprocessor, an approach that aligns with emerging research on novel preprocessing techniques using LLMs [12]. Using a targeted prompt, the LLM extracts a concise summary of the accident’s process and cause, effectively reducing redundant information. Second, to capture fragmented causality and multi-level semantics, the core of FAR-AM utilizes a custom inter-layer self-attention mechanism over BERT. Instead of relying on a single output layer, our model dynamically weights and fuses feature representations from all 12 layers of BERT, allowing it to synthesize information from low-level lexical patterns to high-level contextual understanding. Finally, this fused, feature-rich representation is fed into a TextCNN model, which acts as a powerful feature distiller, identifying the most salient local patterns for accurate classification.

We evaluate FAR-AM on four benchmark datasets: AGNews-title, AGNews-content, THUCNews, and our specialized FIREAR dataset. The experimental findings validate the effectiveness of FAR-AM, demonstrating superior performance over traditional deep learning baselines. Our contributions are as follows:

- We propose a novel hybrid architecture that first uses a prompted LLM to distill critical information from lengthy and noisy fire reports, effectively addressing the challenge of processing overhead and data redundancy.
- We design an inter-layer self-attention mechanism for BERT that dynamically fuses features from all encoder layers. This approach captures a richer, multi-level semantic context, which is crucial for identifying causes from fragmented information scattered throughout a report.
- Our experimental results on four diverse datasets, including the complex, real-world FIREAR dataset, demonstrate that our specialized model significantly outperforms strong baselines, validating its effectiveness for this challenging domain.

## 2 Related work

This section provides an overview of the related work, covering deep learning for text classification, the role of LLMs, and the application of multilayer self-attention mechanisms. While traditional security management has often relied on expert opinions and literature research [13], the analysis of narrative texts from accident reports has become a crucial method for knowledge acquisition and safety improvement in diverse fields such as aviation [14] and railway transportation [15]. Within this context, text classification plays a critical role in extracting key information and identifying accident causes.

### 2.1 Deep learning for text classification

Deep learning has become a powerful tool for text classification, especially in the context of accident analysis [16,17], with applications ranging from chemical incidents [18] to construction accidents [19]. However, the effectiveness of pre-trained models like BERT can diminish when applied to highly specialized domains, where challenges such as class imbalance [20] and the need for complex causal reasoning [21,22] require more tailored approaches. For instance, a recent study by Xiao et al. found that a specialized classical model outperformed a fine-tuned BERT on a complex tourism text dataset, highlighting the profound challenge of domain adaptation [23]. This underscores the need for architectures tailored to specific document types. Concurrently, within the accident analysis domain itself, research is increasingly focused on using machine learning to capture the complex, nonlinear relationships inherent in accident data [24]. While powerful, standard applications of large pre-trained models are not without their own limitations, which have motivated a trend towards exploring more efficient and robust hybrid models that combine the strengths of different architectures [4,25].

### 2.2 LLMs

Recent advancements in LLMs have significantly transformed natural language processing, with numerous studies demonstrating their power in extracting information and analyzing narratives across various domains [26,27]. However, applying monolithic LLMs for end-to-end classification of highly specialized and lengthy documents like fire reports presents its own set of challenges. The computational overhead is substantial, and a general-purpose LLM may struggle to reliably focus on the sparse yet critical evidence required for causation analysis amidst high volumes of noise. Indeed, the application of LLMs to analyze and even generate reports for traffic [28–30] and other incidents [31] is a rapidly growing field, though it often relies on the models’ general knowledge [32]. This has led to an emerging research direction focused on creating hybrid frameworks that combine the strengths of both Small Language Models like BERT and LLMs. A notable example is the FND-LLM framework, which uses an LLM to generate explanatory justifications that enhance a specialized SLM’s detection capabilities in the complex task of fake news detection [33]. This philosophy of using LLMs to augment, rather than replace, specialized models aligns with our approach and is further supported by the success of other hybrid models [25].

### 2.3 Multilayer self-attention mechanism

Multilayer self-attention mechanisms are highly effective for capturing deep contextual relationships in sequence data [34,35], often integrated within architectures like GRU [36] or combined with convolutional layers [37] to enhance feature fusion. The core principle—that a model can weigh the importance of different parts of an input—has proven to be a powerful and versatile tool. This is not limited to text; for instance, Yu et al. successfully applied a self-attention-based architecture to enhance the recognition of complex and noisy bowel sound signals, demonstrating the mechanism’s fundamental strength in extracting both global and local features from challenging, domain-specific data [38]. Despite these advances, many models are not specifically tailored for the textual challenges of accident reports. Our FAR-AM model builds on this principle but is uniquely adapted for our task. Unlike traditional methods that rely on the final output from BERT, FAR-AM’s inter-layer self-attention mechanism is specifically engineered to address the problem of fragmented causality. By dynamically integrating features from all BERT layers, it can synthesize clues from low-level lexical patterns with high-level semantic contexts, facilitating reliable knowledge extraction from uniquely complex documents like fire reports.

## 3 Methodology

### 3.1 Overall framework and procedure

This section details the proposed FAR-AM, a novel hybrid deep learning architecture for the automated classification of fire accident causes. It is important to note that FAR-AM is designed as an adaptive framework with two operational modes depending on the nature of the input text.

- For standard, short-text datasets (e.g., AG News, THUCNews), the framework operates in its core end-to-end mode, which consists of the inter-layer attention-enhanced BERT and the TextCNN classifier. We refer to this core architecture as FAR-AM_core_.
- For long-form, noisy, domain-specific documents (i.e., the FIREAR dataset), the framework incorporates its essential LLM-based preprocessing stage, operating as a complete pipeline. We refer to this full pipeline as FAR-AM.

**Fig 1:**
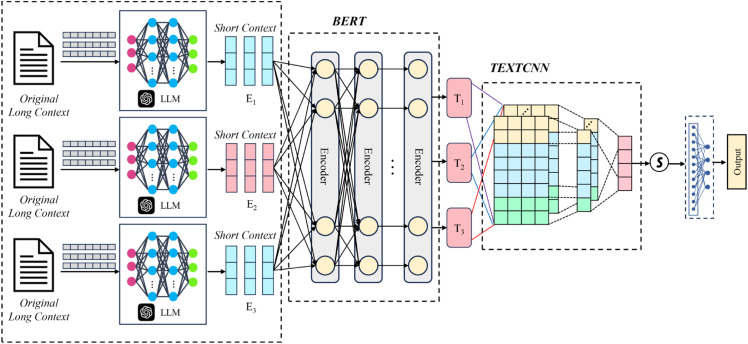
Overall architecture of the FAR-AM model. The input text is first encoded by an LLM to generate short contexts (E1, E2, E3). Then, the context vectors (T1, T2, T3) were generated by the multi-layer Transformer module of BERT. Then, the output vector was convolved and pooled by TextCNN to extract local features and generate feature vectors of fixed size. Finally, after the fully connected layer and Softmax(S), the class probability was output to complete the text classification task.

This adaptability allows the core architecture to be benchmarked on standard tasks while applying its full power to the specialized target domain. The overall architecture of the full pipeline is depicted in Fig 1, and the detailed algorithmic procedure is presented in Algorithm 1.

Algorithm 1 provides a detailed, step-by-step description of the forward pass of the FAR-AM model. The process begins with Stage 1, where the raw, lengthy fire report is passed to the LLM-based preprocessor to generate a concise summary. In Stage 2, this summary undergoes hierarchical feature extraction via the BERT model, where the hidden states from all encoder layers are collected and stacked into a tensor. Stage 3 then performs dynamic feature fusion, where the stacked tensor is processed by our custom inter-layer attention mechanism to produce a single, contextually-rich fused feature matrix. Finally, in Stage 4, this fused matrix is passed to the TextCNN module, which extracts the most salient features and feeds them through a final classification layer to yield the class probability distribution.

**Algorithm 1 The FAR-AM model procedure.**

1: **Input:** A raw, long-text fire report *R*_*long*_.

2: **Parameters:** LLM, BERT model, LayerAttention weights,

TextCNN weights.

3: **Output:** Predicted class probability distribution *P*_*pred*_.

4: **procedure** FAR-AM_Forward*R*_*long*_

5:   // Stage 1: LLM Preprocessing

6:   $S_{short}\leftarrow \text{LLM}(R_{long},\text{prompt})$ ⊳ Generate concise summary

7:   // Stage 2: Hierarchical Feature Extraction

8:   $H_{encoder\_layers}\leftarrow \text{BERT\_Encoder}(S_{short})$ ⊳ Apply Eqs. 1-2

9:   $𝐇\leftarrow \text{Stack}(H_{encoder\_layers})$ ⊳ Apply Eq. 3

10:   // Stage 3: Dynamic Feature Fusion

11:   $H_{fused}\leftarrow \text{LayerAttention}(𝐇)$ ⊳ Apply Eqs. 4-6

12:   // Stage 4: Classification

13:   $\text{logits}\leftarrow \text{TextCNN}(H_{fused})$ ⊳ Apply Eqs. 7-10

14:   $P_{pred}\leftarrow \text{softmax}(\text{logits})$ ⊳ Apply Eq. 11

15:   **return** *P*_*pred*_

16: **end procedure**

### 3.2 Stage 1: LLM-based preprocessing and summarization

A core challenge in analyzing fire reports is their extreme length (often exceeding 12,000 words) and high proportion of narrative noise, which can overwhelm downstream classifiers. To address this, FAR-AM begins with an LLM-based preprocessing stage designed to distill the most salient information.

Specifically, we utilize the Doubao model API as our LLM. Each raw, lengthy fire report is fed into the model with the following targeted prompt:

*“Please output the process and cause of the accident based on the report content, in no more than 512 tokens.”*

This prompt instructs the LLM to act as an expert summarizer, generating a concise summary (maximum 512 tokens) that focuses on the core elements relevant to causation. This resulting summary serves as the direct input for the subsequent feature extraction stages.

### 3.3 Stage 2: Hierarchical feature extraction with BERT

The summarized text from Stage 1 is then processed by a pre-trained BERT model to generate rich, hierarchical semantic representations. The effectiveness of BERT as a powerful baseline for text classification tasks is well-established [39]. Our method leverages the features from all encoder layers of the BERT model.

We denote the input summary as a sequence of tokens $X=\{x_{1},x_{2},\cdots x_{n}\}$. The process begins by converting tokens into initial embedding vectors:

$$H^{\left(0\right)}=[e_{1},e_{2},\cdots ,e_{n}],\;\text{where }e_{i}=\text{TokenEmb}(x_{i})+\text{PosEmb}(i)$$ (1)

Subsequently, these embeddings are processed through the *L* layers of the Transformer encoder (where *L* = 12 for BERT-base). The output of each layer *l* is calculated as:

$${\text{H}}^{\left(l\right)}={\text{TransformerEncoder}}^{\left(l\right)}(H^{\left(l-1\right)})$$ (2)

We collect the outputs from all *L* encoder layers and stack them into a single tensor for feature fusion:

$$H=[{\text{H}}^{\left(1\right)},H^{\left(2\right)},\cdots ,H^{\left(L\right)}]\in {\mathbb{R}}^{n\times L\times d}$$ (3)

where *n* is the sequence length and *d* is the hidden dimension size.

### 3.4 Stage 3: Dynamic feature fusion with inter-layer attention

**Fig 2:**
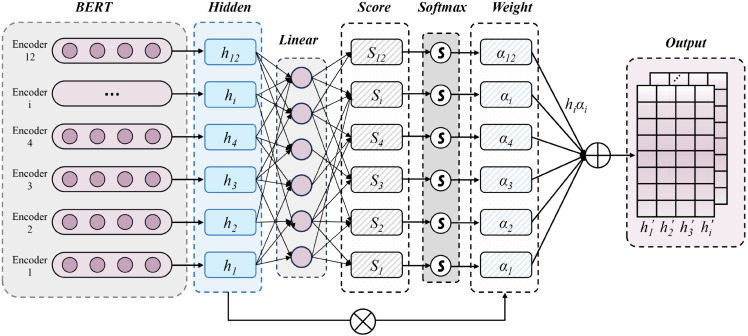
Multi-layer semantic fusion and dynamic attention weighting. Input features $h_{i}^{\left(1\right)},h_{i}^{\left(2\right)},\cdots ,h_{i}^{\left(L\right)}$ are processed through the weight matrix $w_{a}^{\text{T}}$ and a linear transformation to obtain the attention score $s_{i}^{\left(l\right)}$. Then, the Softmax function generates the attention weight $\alpha_{i}^{\left(l\right)}$. By weighting the features with $\alpha_{i}^{\left(l\right)}h_{i}^{\left(l\right)}$ and summing them up, the fused representation is denoted as $H={\left[h_{1},h_{2}\cdots ,h_{n}\right]}^{\text{T}}$.

A foundational premise of our work is that different layers of the BERT encoder capture different granularities of linguistic information. To overcome the limitations of static fusion strategies, we introduce a custom inter-layer self-attention mechanism, building upon the principles of the transformer architecture [40]. This process, which is visually detailed in Fig 2, is a novel contribution of this work.

#### 3.4.1 Attention score and weight calculation.

For each token position *i*, an attention score $s_{i}^{\left(l\right)}$ is calculated for each layer *l*:

$$s_{i}^{\left(l\right)}=w_{a}^{\text{T}}h_{i}^{\left(l\right)}$$ (4)

The scores are normalized across all layers using a softmax function to obtain the attention weights $\alpha_{i}^{\left(l\right)}$:

$$\alpha_{i}^{\left(l\right)}=\frac{\exp(s_{i}^{\left(l\right)})}{\sum_{k=1}^{L}\exp(s_{i}^{\left(k\right)})}$$ (5)

#### 3.4.2 Weighted feature summation and interpretability.

The final, fused representation *h*_*i*_ for each token is the weighted sum of its representations across all layers:

$${\text{h}}_{i}=\sum_{l=1}^{L}\alpha_{i}^{\left(l\right)}h_{i}^{\left(l\right)}$$ (6)

This results in a fused feature matrix $H_{fused}={\left[h_{1},h_{2}\cdots ,h_{n}\right]}^{\text{T}}\in {\mathbb{R}}^{n\times d}$. A significant ancillary benefit of this explicit attention mechanism is enhanced model interpretability.

### 3.5 Stage 4: Classification with TextCNN

The fused feature matrix *H*_*fused*_ is then fed into a TextCNN module for final classification. For each filter with a kernel size of *k*, a convolution operation is applied to produce a feature map *C*_*k*_:

$$c_{k,i}=\text{ReLU}({𝐖}_{k}\cdot H_{fused}[i:i+k-1]+b_{k})$$ (7)

A max-over-time pooling operation is then applied to each feature map:

$$p_{k}=\max(C_{k})$$ (8)

The outputs from all filters are concatenated to form the final feature vector:

$$P_{cat}=[p_{1},p_{2},\ldots ,p_{m}]$$ (9)

This vector is then passed through a fully connected layer to produce the final logits:

$$\text{logits}={𝐖}_{fc}\cdot P_{cat}+b_{fc}$$ (10)

### 3.6 Model training and optimization

The entire FAR-AM model is trained end-to-end. The final prediction probabilities are obtained by applying a softmax function to the logits:

$$\hat{y}=\text{softmax}(\text{logits})$$ (11)

We use the cross-entropy loss function to optimize the model parameters:

$$\text{L}(\hat{y},y)\text{=-}\sum_{i=1}^{N}\sum_{j=1}^{C}y_{i}^{j}\text{log(}{\hat{y}}_{i}^{j})$$ (12)

where *y* is the ground-truth label, *N* is the number of training samples, and *C* is the number of classes.

## 4 Experimental setup

### 4.1 Datasets

To comprehensively evaluate the performance and generalization capabilities of the FAR-AM model, we conducted experiments on four benchmark datasets. These datasets were specifically chosen to assess the model’s effectiveness across different languages (English and Chinese) and text lengths (long and short corpora), ensuring a robust validation. The detailed summary statistics for all datasets, including our specialized FIREAR corpus and a breakdown of its composition, are presented in Table 1.

**Table 1:** Summary statistics of evaluation datasets.

Dataset	#Docs	Avg. Length	#Classes
**Public Datasets**			
AG News (content)	12760	44.03	4
THUCNews	6500	15.25	10
AG News (title)	12760	12.04	4
**Our Proposed Dataset (FIREAR)**			
*Overall*	*1700*	*479.82* ^a^	*4*
*Class Distribution:*			
- Violation of construction regulations (VCR)	560	-	-
- Electrical malfunction (EM)	405	-	-
- Improper use of flammable materials (IUFEM)	398	-	-
- Improper use of open flames (IUOF)	337^b^	-	-

^a^ Average length after summarization by LLM. The original average length is 10,351 words. ^b^ This category includes 187 original reports and 150 samples generated by an LLM for data augmentation.

Long Corpus Datasets.

(1) AG News dataset(content): The AG News (Antonio Gulli’s News) dataset is a public English news corpus for text classification [41]. It contains four categories of news and a total of 120,000 training samples and 7,600 test samples. We only use 10% of the data in our experiments.

(2) FIREAR dataset: The FIREAR dataset is a specialized Chinese-language corpus developed for this study, comprising 1,700 fire accident investigation reports. Of these, 1,550 reports were collected from publicly available documents issued by various levels of Emergency Management Departments in China between 2015 and 2025. To address class imbalance, we augmented the ‘improper use of open flames’ category with an additional 150 samples generated using the Doubao LLM. To further enhance transparency, one representative, public-domain report from each category is available at https://github.com/javalivepeng/FIREAR-sample.

Short Corpus Datasets.

(1) AG News dataset(title):This dataset is derived from the titles of news articles in the AG News dataset, with an average length of 12.04 characters and is categorized into four classes.

(2) THUCNews dataset: The THUCNews (Tsinghua University Chinese News) dataset is a public Chinese news corpus for text classification. The dataset uses 10 categories, each with 6,500 items. We only use 10% of the data. The dataset is split as follows: training set: 500×10, validation set: 50×10, test set: 100×10 [42,43].

AG News dataset, THUCNews dataset and FIREAR dataset represent long text English dataset, short text Chinese dataset and long text Chinese dataset, respectively. By selecting cross-language and cross-length datasets, we can evaluate the model’s performance on diverse data, ensuring a more comprehensive assessment.

### 4.2 Hyper-parameter settings

The values of hyper-parameters for our models are shown in Table 2. We use the Transformer to develop and train BERT models in our experiments. For all experiments, we fine-tune the model with the AdamW optimizer and a learning rate of 1e-5. The hidden size of the BERT model is configured to 768, and a dropout rate of 0.2 is applied to both word and character embeddings. We conducted experiments using convolution kernels with sizes of 3, 4, and 5, and quantities of 2, 3, 5, 10, and 20. The batch size was 16 and the model was fine-tuned on the individual tasks for 20 rounds.

**Table 2:** Hyper-parameter values.

Parameter	Value	Parameter	Value
learning rate *lr*	1e-5	character dropout	0.2
BERT hidden	768	batch_size	16
kernel size	3,4,5	number of kernels	2,3,5,10,20
word dropout	0.2	epoch	20

### 4.3 Baselines

To evaluate our model’s performance in text classification, we first preprocess our collected data using the LLMs to exclude redundant and interfering information in the data. Then we use the following models as the baseline models of the experiment to provide a reference effect for subsequent models.

**word2vec-textcnn** [44] represents a classical deep learning approach that combines static word embeddings with a convolutional network. It first utilizes a pre-trained Word2Vec model to convert input words into dense vector representations. These vectors are then fed into a standard TextCNN architecture, which extracts local n-gram features to perform the final classification. This model serves as a strong baseline to evaluate the benefits of contextualized embeddings over static ones.

**RoBERTa** [45] is an optimized version of BERT that improves upon its pre-training strategy. Key modifications include training on a much larger dataset, removing the next sentence prediction task, and dynamically changing the masking pattern. Due to these enhancements, RoBERTa often achieves better performance than BERT on various downstream NLP tasks and serves as a highly competitive baseline.

**BERT** [39] is a bidirectional deep learning-based model that processes text from both the left and right sides, rather than from a single direction. The most recent version of BERT employs the Transformer architecture, which is made up of several encoding layers. It has been shown to be effective in a wide range of computational tasks, including inference, semantic understanding, NLP, text segmentation, and classification.

**TEXT-CNN** [46] is a deep learning model for text classification based on Convolutional Neural Networks. It extracts n-gram features through a one-dimensional convolution layer to capture the local information of the text, and uses convolution kernels of multiple sizes to obtain different ranges of features. It combines the max pooling layer to extract important information, and then classifies through the fully connected layer, which is especially suitable for short text classification tasks.

**TEXT-RNN** [47] is a text classification model based on Recurrent Neural Network. Text sequences are usually processed using LSTM or GRU to progressively capture sequential information and contextual relationships in sentences. Different from CNN, TEXT-RNN is more suitable for processing long text tasks and can effectively retain global dependency information, which is widely used in sentiment analysis and text classification.

**DPCNN** [48] is a text classification model implemented by stacking deep convolutional structures. It uses region embedding to represent text features, and combines narrow convolutional layers and shortcut connections to build a deep pyramid structure. The design is able to capture long-distance dependencies while maintaining efficient computation, which is a significant advantage in long text classification tasks.

### 4.4 Overview of evaluation metrics

In this study, we use several important evaluation metrics to assess the performance of the FAR-AM model in text classification tasks, including Precision, Recall, F1 Score, and Accuracy. These metrics provide a comprehensive understanding of the model’s performance, and their definitions and calculation methods are as follows:

**(1) Precision**: Precision is the ratio of true positive samples to the total predicted positive samples. It measures the accuracy of the model, especially important in scenarios where minimizing false alarms is critical. The formula for precision is:

$$\text{Precision}=\frac{TP}{TP+FP}$$ (13)

where *TP* (True Positive) represents the number of true positive samples, and *FP* (False Positive) represents the number of false positive samples.

**(2) Recall**: Recall is the ratio of true positive samples to the total actual positive samples. It reflects the model’s ability to identify all relevant instances, particularly important when the goal is to cover all positive samples. The formula for recall is:

$$\text{Recall}=\frac{TP}{TP+FN}$$ (14)

where *FN* (False Negative) represents the number of false negative samples.

**(3) F1 Score**: The F1 Score is the harmonic mean of precision and recall, providing a balance between the two metrics. It is particularly useful in scenarios with class imbalance, and its calculation is given by:

$$\text{F1}=2\times \frac{\text{Precision}\times \text{Recall}}{\text{Precision}+\text{Recall}}$$ (15)

**(4) Accuracy**: Accuracy measures the ratio of correctly classified samples to the total samples. It is one of the most commonly used evaluation metrics for classification models, especially when the classes are balanced. The formula for accuracy is:

$$\text{Accuracy}=\frac{TP+TN}{TP+TN+FP+FN}$$ (16)

where *TN* (True Negative) represents the number of true negative samples.

In this study, we primarily selected accuracy as the evaluation metric for our experiments because it offers a clear measure of the proportion of correctly classified samples. This straightforward indicator allows us to effectively assess the performance of the FAR-AM model across various datasets. Through these evaluation metrics, we can gain a comprehensive understanding of the classification effectiveness of FAR-AM and highlight its advantages.

## 5 Experimental results

### 5.1 Performance comparison

We evaluated the performance of our FAR-AM framework against a diverse set of strong baseline models across four datasets. The comprehensive results for accuracy and F1-score are presented in Table 3. For the public datasets (AG News and THUCNews), all models were applied directly to the provided texts. For the complex FIREAR dataset, we followed standard practice by applying the baseline models to the raw, truncated reports, while our proposed FAR-AM was utilized as a complete pipeline, including its LLM preprocessing stage, to demonstrate its full system-level effectiveness.

**Table 3:** The results of accuracy and F1 on document classification with different models.

Model	AG News (title)		AG News (content)		THUCNews		FIREAR^a^	
	accuracy	F1	accuracy	F1	accuracy	F1	accuracy	F1
TEXT-CNN	0.7655	0.7821	0.7870	0.7772	0.8781	0.8544	0.6081	0.5776
word2vec-textcnn	0.7220	0.7012	0.7871	0.7913	0.7563	0.7443	0.5954	0.5812
TEXT-RNN	0.7760	0.7842	0.8176	0.8021	0.2816	0.2604	0.6023	0.5742
BERT	0.8142	0.8028	0.8600	0.8642	0.9428	0.9215	0.6377	0.6235
BERT-DPCNN	0.7009	0.6877	0.8224	0.8120	0.9085	0.8892	0.6912	0.6725
RoBERTa	0.8523	0.8443	0.8658	0.8602	0.9332	0.9221	0.7028	0.6723
BERT-TEXTCNN	0.8011	0.8002	0.8459	0.8413	0.9328	0.9206	0.6752	0.6513
**FAR-AM**_core_ **(ours)**	**0.8630**	0.8619	**0.8741**	0.8792	**0.9448**	0.9328	-	-
**FAR-AM (ours)**	-	-	-	-	-	-	**0.7358**	0.7065

^a^ For the FIREAR dataset, all baseline models were run on raw reports. The FAR-AM result reflects the full pipeline including the LLM preprocessing stage.

On the public benchmark datasets, our core architecture, FAR-AM_core_, consistently demonstrated superior performance. Across the AG News (title), AG News (content), and THUCNews datasets, FAR-AM_core_ achieved the highest accuracy and F1-scores, outperforming all baseline models. Notably, it surpassed the powerful RoBERTa baseline on AG News (content) (0.8741 vs. 0.8658 accuracy) and THUCNews (0.9448 vs. 0.9332 accuracy), which indicates that our core architecture, featuring the inter-layer attention mechanism, is a robust and highly effective general-purpose text classifier.

The most significant results were observed on our specialized, long-form FIREAR dataset. Here, the full FAR-AM pipeline achieved an accuracy of 73.58% and an F1-score of 70.65%, substantially outperforming all other methods. It surpassed the strongest baseline, RoBERTa (70.28% accuracy), by a margin of over 3.3 percentage points. This large performance gap validates the effectiveness of our end-to-end framework. It highlights that for long-form, noisy, domain-specific text, standard advanced models like BERT and even the powerful RoBERTa struggle when applied directly, whereas our FAR-AM pipeline, with its intelligent LLM summarization and hierarchical feature fusion, provides a markedly superior solution.

In summary, the results demonstrate the dual strengths of our work: the FAR-AM_core_ architecture is a powerful classifier excelling on standard benchmarks, and the complete FAR-AM pipeline is a highly effective, specialized solution for complex, real-world document analysis tasks where other models fall short.

Table 3 presents the performance of various models on document classifi-cation tasks across different datasets, including AG News (title), AG News(content), THUCNews and FIREAR. The FAR-AM model performs excep-tionally well, achieving the highest accuracy and F1 scores across all datasets.In the AG News (title) dataset, which includes short English texts, FAR-AM reached an accuracy of 86.30% and an F1 score of 86.19%. This effectiveness is due to the model’s capability to quickly extract key information using the BERT framework and self-attention mechanisms. For the AG News (content) dataset, FAR-AM achieved an accuracy of 87.41% and an F1 score of 87.92%, demonstrating its adaptability to longer texts. In the Chinese datasets, FAR-AM secured an accuracy of 94.48% and an F1 score of 93.28% on the THUCNews dataset, illustrating its strength in short text classifica-tion. In the FIREAR dataset, which contains long Chinese texts, the model attained an accuracy of 73.58% and an F1 score of 70.65%, indicating its abil-ity to handle complex documents. Overall, the results highlight FAR-AM’s effectiveness in document classification across various datasets and contexts.

Our method incorporates LLMs to enhance the understanding and extrac-tion of key information. The inter-layer self-attention mechanism of BERT dynamically fuses semantic information from different layers. The approach improves the model’s ability to represent complex texts and enhances classi-fication performance.

### 5.2 Ablation study

To rigorously dissect the contribution of each key component within the FAR-AM architecture, we conducted a comprehensive series of ablation experiments on the FIREAR dataset. By systematically removing or replacing core modules with simpler alternatives, we can quantify their impact on the model’s overall performance. The results are presented in Table 4, which compares the full FAR-AM model against several ablated configurations.

**Table 4:** Results of the ablation study on the FIREAR dataset.

Model/Configuration	Accuracy
FAR-AM (Full Model)	0.7358
w/o LLM	0.4667
w/o Inter-layer Attention (use Simple Average)	0.6929
w/o Inter-layer Attention (use Last Layer)	0.6482
w/o TextCNN	0.6381

^*^ w/o stands without.

The experimental results clearly demonstrate that every component of our proposed model makes a significant and indispensable contribution to its final performance. The key findings are as follows:

1. The Critical Role of LLM Preprocessing: Comparing the full FAR-AM model with the ‘w/o LLM‘ configuration, where the model was fed with raw, truncated report text, reveals a dramatic plummet in accuracy from 0.7358 to 0.4667. This substantial decrease of nearly 27 percentage points underscores the severity of the noise and redundancy in the original long-form reports. It provides powerful evidence that the LLM-based preprocessing is a critical component for enabling the downstream model to effectively process the complex data.

2. The Superiority of the Attention-based Fusion Mechanism: We compared our inter-layer attention mechanism against two simpler fusion strategies. When replacing our dynamic attention with a simple averaging of all 12 encoder layers (‘w/o Inter-layer Attention (use Simple Average)‘), the accuracy dropped to 0.6929. When relying only on the final (12th) layer of BERT (‘w/o Inter-layer Attention (use Last Layer)‘), the accuracy fell even further to 0.6482. These results strongly validate the core hypothesis of our paper: that dynamically weighting and fusing features from the entire hierarchy of BERT layers is substantially more effective than either naive fusion or the common practice of using only the final layer’s output.

3. The Effectiveness of the TextCNN Classifier: Removing the final TextCNN module (‘w/o TextCNN‘) resulted in a significant performance drop to 0.6381. This indicates that the TextCNN acts as a powerful and necessary feature distiller, effectively capturing the most salient local patterns from the feature-rich representation created by the attention fusion stage.

In summary, the ablation study confirms that the superior performance of FAR-AM is not attributable to a single component, but to the synergistic interplay of all three stages: intelligent summarization by the LLM, sophisticated hierarchical feature fusion by the inter-layer attention, and effective feature distillation by the TextCNN.

## 6 Analysis

### 6.1 Training loss and accuracy progression

**Fig 3:**
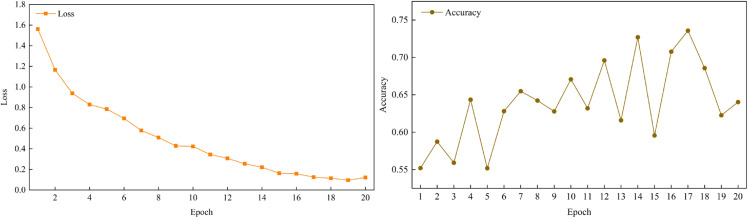
Training loss and accuracy progression over epochs.

To measure the model’s performance during the training process, we monitored both the loss and accuracy across training rounds on the FIREAR dataset. The experimental results are shown in Fig 3.

As depicted in Fig 3 (left), the training loss exhibits a smooth and consistent downward trend, gradually decreasing over the epochs and stabilizing around 0.12, which indicates that the model is effectively learning and converging.

The accuracy curve, shown in Fig 3 (right), demonstrates a clear overall upward trend, beginning to stabilize after approximately 16 rounds and reaching a peak value of 0.7358. The fluctuations observed in the validation accuracy curve are a common phenomenon when fine-tuning large pre-trained models on specialized, smaller datasets like FIREAR. This volatility can be attributed to the variance between mini-batches and the model’s high sensitivity to parameter updates during the complex optimization process. Despite these fluctuations, the consistent upward trajectory and the stabilization at a high-performance level confirm the model’s robust learning capability.

### 6.2 Number and size of convolution kernels

**Fig 4:**
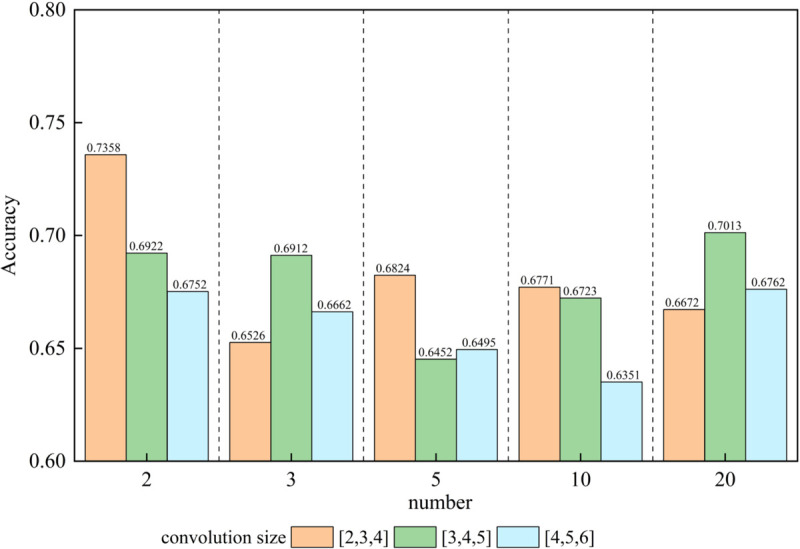
The effect of the number and size of convolution kernels on the model.

We analyze the impact of varying the number and size of convolution kernels on model performance. Fig 4. illustrates the effect of different number and size of convolution kernels.

#### 6.2.1 Effect of varying the number of convolution kernels.

We analyze how changing the number of convolution kernels affects model performance. When the size of the convolution kernel is set to 3, increasing the number of kernels from 2 to 3 results in a significant drop in performance, with accuracy decreasing from 0.7358 to 0.6526. As we continue to increase the number of convolution kernels beyond three, the performance stabilizes, indicating a point of diminishing returns in terms of accuracy. This suggests that there is an optimal range for the number of kernels beyond which additional kernels do not enhance performance and may even degrade it.

#### 6.2.2 Effect of varying the size of convolution kernels.

Next, we examine the impact of varying the size of convolution kernels on model performance. For kernel sizes of [2, 3, 4], the performance remains relatively stable at approximately 0.6830 regardless of the number of kernels used. This observation points to the effectiveness of these specific kernel sizes in achieving both feature extraction and generalization regardless of the number of kernels. Conversely, when the kernel size is increased to [4, 5, 6], we observe a decline in performance from 0.6830 to 0.6604 as the number of kernels increases, followed by a slight recovery. This indicates that larger kernels may lose effectiveness in capturing local features when excessive kernels are employed due to redundancy or overfitting.

Our findings suggest that the optimal configuration for convolution kernels is two kernels with sizes of [2, 3, 4]. This configuration balances efficient feature extraction and generalization while minimizing the risk of overfitting and redundancy. The results indicate that smaller kernels effectively capture local features, but their performance may degrade when their number increases beyond an optimal point.

### 6.3 Performance over different categories of FIREAR datasets

To evaluate the performance of the FAR-AM model in different safety risk detection tasks, we analyze its performance across four categories: improper use of open flames, violation of construction regulations, improper use of flammable and explosive materials, and electrical malfunction. The results are shown in Table 5. The model demonstrates strong performance in detecting illegal construction and electrical faults, achieving accuracy rates of 0.8671 and 0.8378, respectively. At the same time, it also maintains a high recall rate and F1 score in two categories, which are 0.87 and 0.85, and 0.85 and 0.76, respectively. These results indicate that the model can effectively identify these two types of security risks. However, in the detection of improper use of open flame, the performance of the model is not satisfactory, with an accuracy of only 0.6905, and the precision and recall are also relatively low, with 0.64 and 0.69, respectively, resulting in an F1 score of only 0.67. This result stems from the limited number of samples in this category, which hinders the model’s ability to learn an effective feature representation. For improper use of flammable and explosive items, the model shows moderate performance, with an accuracy of 0.6822, precision of 0.79, recall of 0.68, and an F1 score of 0.66, suggesting room for improvement in identifying such risks. Overall, the FAR-AM model shows good performance in the detection of illegal construction and electrical faults, but further optimization and more data support are needed in the detection of improper use of open flame. It is important to note that while the absolute accuracy has room for improvement, the reported performance signifies a substantial relative advancement over baseline models on this highly complex and noisy dataset.

**Table 5:** Performance of FAR-AM models on different data categories.

category	accuracy	precision	recall	F1
IUOF	0.6905	0.64	0.69	0.67
VCR	0.8671	0.72	0.87	0.85
IUFEM	0.6822	0.79	0.68	0.66
EM	0.8378	0.75	0.85	0.76

Performance of FAR-AM models on different data categories. IUOF represents improper use of open flames. VCR represents violation of construction regulations. IUFEM represents improper use of flammable and explosive materials. EM represents electrical malfunction.

### 6.4 Comparison with standalone LLMs

To further contextualize the performance of our specialized FAR-AM framework, we also evaluated the zero-shot classification capabilities of several general-purpose LLMs on the FIREAR dataset. This experiment was designed to assess whether a large, monolithic model could achieve comparable performance without a specialized architecture when confronted with the raw, complex source documents.

#### 6.4.1 Experimental methodology.

The evaluation was conducted as a zero-shot text classification task. We provided each LLM with the full-text original accident reports from the FIREAR dataset, which required the models to process texts with an average length of over 10,000 words. The interaction with each model was performed via its respective official API. The LLMs were instructed to classify the report into one of the four predefined fire cause categories using the following prompt template:

*“As an expert fire investigator, Your task is to classify the cause of a FIREAR. Please choose only one of the following four categories: improper use of open flames, violation of construction regulations, improper use of flammable and explosive materials, electrical malfunction. Please output the category name. Report Text: [full fire report text]*”

The models evaluated were ChatGPT, DeepSeek, Doubao, and Wenxin Yiyan. Accuracy was calculated based on an exact string match between the model’s output and the ground-truth category name. Any response containing additional text, or failing to exactly match one of the predefined category strings, was considered an incorrect classification.

#### 6.4.2 Results and analysis.

The results of this comparison are presented in Table 6. The findings indicate that while modern LLMs possess foundational capabilities for this task, their zero-shot performance is significantly hampered when processing long-form, noisy, real-world documents directly. ChatGPT achieved the highest accuracy among the tested LLMs at 62.06%.

**Table 6:** Zero-shot classification accuracy of standalone LLMs on the full-text FIREAR dataset.

Large Language Model	Accuracy
ChatGPT	0.6206
DeepSeek	0.6028
Doubao	0.5532
Wenxin Yiyan	0.5048

Notably, this result is significantly lower than the 73.58% accuracy achieved by our specialized FAR-AM framework. This performance gap of over 11 percentage points underscores a crucial finding of our work: for complex, domain-specific classification tasks like fire accident analysis, a specialized hybrid architecture that includes an intelligent preprocessing stage (like FAR-AM) is more robust and effective than simply applying a general-purpose, monolithic LLM to the raw text. The result suggests that even powerful LLMs can struggle to distill the core causal signals from thousands of words of noise, validating the necessity of our multi-stage pipeline approach.

## 7 Discussion

Our experimental results indicate a competitive performance for the FAR-AM framework, particularly on the complex FIREAR dataset. This section aims to interpret the implications of these numerical results, discuss the strengths and limitations of our approach, and contextualize our findings within the wider landscape of NLP and accident analysis research.

### 7.1 Performance and architectural validation

The quantitative results of our model lead to several findings. Firstly, the performance of FAR-AM on the FIREAR dataset provides support for our central hypothesis: for highly complex, domain-specific classification tasks, a specialized hybrid architecture may be more effective than both standard fine-tuned models like BERT or RoBERTa and general-purpose LLMs applied directly to raw text. This suggests potential limitations for monolithic approaches when faced with the combined challenges of extreme document length, semantic noise, and fragmented causality.

Secondly, the ablation study reveals the important role of each stage in our pipeline. The notable drop in accuracy when the LLM preprocessor was removed suggests that for such lengthy and noisy documents, an intelligent, semantic-level preprocessing step is highly beneficial. This supports the value of a coarse-to-fine framework. The LLM acts as a “coarse” filter, identifying the most relevant sections of the text, while the subsequent BERT with inter-layer attention performs the “fine-grained” analysis on this distilled information. This two-stage paradigm presents a promising strategy that could be generalized to other long-document analysis domains.

Furthermore, our analysis of performance across different categories on the FIREAR dataset provides a more nuanced view. The model achieved strong performance on categories with more distinct linguistic patterns, such as ‘violation of construction regulations’ (VCR) and ‘electrical malfunction’ (EM). Conversely, its performance was relatively lower on categories like ‘improper use of open flames’ (IUOF), which can be described in more varied and ambiguous language. This indicates that while our model is effective, its performance is still influenced by the inherent separability of the classes’ textual features, highlighting an area for future improvement.

### 7.2 Strengths and limitations of the FAR-AM framework

Our study presents a framework with several notable strengths. A key strength is its purpose-built design, which systematically deconstructs the problem of complex document classification. Another strength is its demonstrated robustness and adaptability. The framework’s competitive performance on both the noisy, domain-specific FIREAR dataset and the clean, general-purpose public datasets (via FAR-AM_core_) suggests its versatility.

An additional benefit is the framework’s potential for model interpretability. The inter-layer attention mechanism allows for an analysis of the learned layer weights, offering a window into which levels of semantic abstraction BERT finds most useful for this task. For a brief case study, consider one of the sample reports for ‘electrical malfunction’ made publicly available in the project’s GitHub repository. The LLM preprocessor would distill the multi-page report to a summary highlighting phrases like ‘short circuit’ or ‘faulty wiring’. Subsequently, the inter-layer attention mechanism would likely assign high weights to the BERT layers that best capture the technical meaning of these terms. This provides a tangible, albeit simplified, example of the model’s logical flow, a valuable feature for applications in safety-critical domains.

However, we also acknowledge several limitations. Firstly, the performance of the full FAR-AM pipeline is inherently dependent on the quality of the upstream LLM summarizer. Secondly, as noted above, the model’s performance is still sensitive to class imbalance and the inherent ambiguity of certain categories. Lastly, the multi-stage nature of the pipeline introduces greater computational complexity compared to a single end-to-end model.

### 7.3 Implications for NLP and accident analysis research

Our work is informed by and aims to contribute to several emerging trends in NLP research. The hybrid nature of FAR-AM aligns with a growing body of work that combines the strengths of different models to solve complex problems [25,33]. Our approach of using an LLM for strategic preprocessing, rather than end-to-end classification, represents a contribution to this trend, demonstrating a potential pathway to harness the power of LLMs in a more controlled and efficient manner.

Furthermore, our findings on the domain-specific FIREAR dataset resonate with studies in other specialized fields. For instance, the observation by Xiao et al. that a specialized model can outperform a powerful, general-purpose BERT on a niche tourism dataset reinforces our conclusion that domain adaptation remains a significant challenge [23]. Within the field of accident analysis, our work extends the application of advanced deep learning. While studies like Liang et al. have effectively used machine learning to model nonlinear relationships in structured accident data [24], our framework offers an approach for modeling the deep causal semantics embedded within lengthy, unstructured textual reports, which we hope is a useful contribution to the field.

## 8 Conclusion and future work

In this work, we proposed and validated a novel text classification model, FAR-AM, which effectively addresses the challenge of automated cause-of-accident classification from complex fire reports by dynamically fusing multi-layer semantic features from BERT. Our experimental results not only confirm the superiority of our model over several strong baselines but also yield a crucial insight: for complex, domain-specific classification tasks such as accident analysis, a specialized hybrid architecture engineered to handle specific textual features can be more robust and effective than a general-purpose, monolithic LLM. This finding underscores the continued value of targeted model design in an era increasingly dominated by large-scale pre-trained models, highlighting that architectural innovation remains a key driver of performance in high-stakes, specialized domains. This work contributes both a tangible tool for enhancing fire safety management and a methodological blueprint for similar analytical tasks.

Future work will focus on several key areas to build upon the findings of this study. Firstly, to address performance limitations on underrepresented classes, more advanced data augmentation and few-shot learning strategies could be explored. Secondly, the framework’s computational complexity could be addressed through model distillation or quantization to create a more lightweight version of FAR-AM for practical deployment. Thirdly, the model’s interpretability could be further enhanced by developing a visualization tool for the inter-layer attention weights to provide investigators with actionable insights into the model’s decision-making process. Finally, evaluating the adaptability of the FAR-AM framework to other types of long-form, unstructured accident reports, such as those from the aviation or chemical industries, will be a valuable step in assessing its broader applicability.
